# Getah Virus Infection among Racehorses, Japan, 2014

**DOI:** 10.3201/eid2105.141975

**Published:** 2015-05

**Authors:** Manabu Nemoto, Hiroshi Bannai, Koji Tsujimura, Minoru Kobayashi, Takuya Kikuchi, Takashi Yamanaka, Takashi Kondo

**Affiliations:** Japan Racing Association, Shimotsuke, Japan (M. Nemoto, H. Bannai, K. Tsujimura, T. Yamanaka, T. Kondo);; Japan Racing Association, Miho, Japan (M. Kobayashi, T. Kikuchi)

**Keywords:** Getah virus, horse, outbreak, vaccine, Japan, viruses, vector-borne infections, mosquitoes, Alphavirus

## Abstract

An outbreak of Getah virus infection occurred among racehorses in Japan during September and October 2014. Of 49 febrile horses tested by reverse transcription PCR, 25 were positive for Getah virus. Viruses detected in 2014 were phylogenetically different from the virus isolated in Japan in 1978.

Getah virus (genus *Alphavirus*, family *Togaviridae*) is a mosquito-borne virus that was first isolated in Malaysia in 1955 from *Culex* spp. mosquitoes (*1*). Serologic evidence suggests that Getah virus is widespread from Eurasia to Australasia (*1*,*2*). In horses, the virus causes fever, rash on the body, and edema in the legs (*2*); the virus is also pathogenic to pig fetuses and newborn piglets (*3*,*4*) and can cause fever in humans (*5*). Outbreaks of Getah virus infection have occurred among horses in 1978, 1979, and 1983 in Japan (*2*) and in 1990 in India (*6*).

An inactivated whole-virus vaccine (Nisseiken Co., Ltd, Tokyo, Japan) is available to prevent Getah virus infection and is mainly administered to thoroughbred racehorses registered by the Japan Racing Association. It is recommended that this vaccine be administered twice in the first year of registration (mainly 2-year-old horses) and then annually as a booster before each mosquito season. This vaccine contains the MI-110 strain isolated from a febrile horse during the outbreak in 1978.

In mid-September 2014, the number of febrile horses began to increase at the Miho Training Center of the Japan Racing Association in Ibaraki Prefecture; we identified Getah virus infection among these horses. The outbreak of Getah virus infection in 1978 occurred at this facility. We summarize the epidemiologic features and molecular characterization of the epidemic virus.

## The Study

Approximately 2,000 horses are stabled at the facility in which this outbreak occurred. In September and October 2014, a total of 36 and 39 horses, respectively, became febrile. The numbers of pyretic horses in September and October during 2009–2013 were 16.0 ± 4.4 and 17.6 ± 1.7 (mean number ± SD), respectively.

EDTA-treated blood samples and nasal swab samples from 49 and 48 pyretic horses, respectively, were collected during September 25–November 2. Paired serum samples were collected from 19 febrile horses during the acute (September 15–October 12) and convalescent (2–4 weeks later) phases. Nasal swab samples were suspended in 2.5 mL of transport medium (*7*). Viral RNA and DNA were extracted from the blood samples and nasal swab samples by using a nucleic acid isolation kit (MagNA Pure LC Total Nucleic Acid Isolation Kit, Roche Diagnostics, Mannheim, Germany). Reverse transcription PCR (RT-PCR) was conducted using a primer pair targeting nonstructural protein 1 (NSP1) of Getah virus (OneStep RT-PCR Kit, QIAGEN, Hilden, Germany) by using the RNA extracted from the blood samples (*8*). PCR was also used to detect the specific genes of equid herpesviruses 1 and 4 within the blood samples (*9*). Reverse transcription loop-mediated isothermal amplification was used to detect equine influenza virus in the nasal swab samples (*7*). Equine arteritis virus was tested by real-time RT-PCR by using the blood samples (*10*). A virus neutralization test for Getah virus was conducted on Vero cells using the MI-110 strain, which was isolated in 1978 (*11*) and is the current vaccine strain, as described previously (*12*) with slight modification. The neutralizing antibody titers were determined as the reciprocal of the highest serum dilution that inhibited viral cytopathic effects. Seroconversion was defined as >4-fold increase in the antibody titer between paired serum samples. Serum collected from horses vaccinated after August 1 were not used in this study because the neutralization test cannot distinguish an increase in antibodies induced by natural infection from that induced by vaccination.

The NSP1 and capsid genes in the RT-PCR–positive samples (strain designation Miho-2014) and MI-110 strain were amplified by using previously described primer pairs (*8*) and were sequenced commercially (Fasmac Co., Ltd., Atsugi, Japan). Sequences were analyzed with the Vector NTI Advance 11 software (Invitrogen, Carlsbad, CA, USA). Phylogenetic analyses of the nucleic acid sequences were conducted with MEGA 5.2 software (*13*). Phylogenetic trees based on the NSP1 and capsid genes were constructed by using the neighbor-joining method. The statistical analysis of the trees was conducted with the bootstrap test (1,000 replicates). The accession numbers registered in GenBank/EMBL/DDBJ are as follows: the partial sequences of the NSP1 gene Miho-2014 (LC012885) and MI-110 (LC012887); and the partial sequences of the capsid gene Miho-2014 (LC012884) and MI-110 (LC012886).

Of the 49 blood samples tested among horses 2–7 years of age, 25 were positive for Getah virus by RT-PCR (Table). All blood samples were negative for equid herpesviruses 1 and 4 and equine arteritis virus, and all nasal swab samples were negative for equine influenza virus. In the neutralization test, 16 of 19 paired serum samples showed seroconversion (>4-fold increase) to the MI-110 strain of Getah virus (Table). In total, 33 febrile horses were positive for Getah virus infection by RT-PCR, neutralization test, or both. Seventeen horses had edema in their legs, and 4 had rashes on their bodies; these horses constituted a small percentage of febrile horses. Getah virus randomly infected horses stabled throughout the Miho Training Center. All the horses that were positive for Getah virus infection recovered after treatment of signs.

**Table:** Number of newly febrile horses and results of RT-PCR and a virus neutralization test, Japan, 2014*

Test	Sep 1–7, n = 3	Sep 8–14, n = 3	Sep 15–21, n = 12	Sep 22–28, n = 15	Sep 29–Oct 5, n = 13	Oct 6–12, n = 16	Oct 13–19, n = 7	Oct 20–26, n = 2	Oct 27–Nov 2, n = 6
RT-PCR	NT	NT	NT	4/5†	11/13	6/16	3/7	1/2	0/6
Neutralization	NT	NT	4/4‡	4/4	5/5	3/6	NT	NT	NT

*NT, not tested; RT-PCR, reverse transcription PCR. †Number of positive samples/number of blood samples examined. ‡Number of seroconversions/number of paired serum samples examined.

The first and last samples that were positive by neutralization test, RT-PCR, or both were collected from pyretic horses on September 15 and October 25, respectively. These results show that the Getah virus infection occurred among racehorses at the Miho Training Center from mid-September through late October 2014.

We analyzed the sequences of the NSP1 and capsid genes of 10 positive samples and the MI-110 strain. The nucleic acid sequences of the NSP1 (381 bp) and capsid (552 bp) genes were completely identical among the 10 Getah viruses detected in 2014. The nucleic acid sequence identities between the Getah virus detected in 2014 and MI-110 were 98.7% for the NSP1 gene and 99.1% for the capsid gene. Phylogenetic analyses were performed with the nucleic acid sequences of the Getah virus NSP1 and capsid genes, including those of isolates from horses, mosquitoes, and pigs (Figure), and showed that the Getah viruses detected in 2014 clustered apart from the MI-110 strain.

**Figure:**
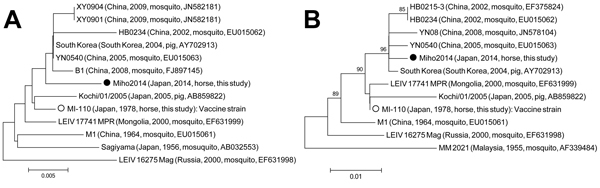
Phylogenetic analyses of the nucleotide sequences of the (A) nonstructural protein 1 (NSP1) gene (nt 218–598) and (B) capsid gene (nt 7645–8196) of Getah virus isolated in Japan, 2014. The genome positions of the NSP1 and capsid genes correspond to those of Kochi/01/2005 strain (GenBank accession no. AB859822) (*14*). Closed and open circles represent Miho2014, the strain isolated in this study, and MI-110, the strain isolated in 1978, respectively. The percentage bootstrap support is indicated by the value at each node; values <70 are omitted. Scale bars indicate nucleotide substitutions per site.

## Conclusions

The first outbreak of Getah virus infection among racehorses occurred in September–November 1978, and another outbreak in 2014 occurred around the same period at the same facility. In 1978, of 1,903 stabled horses, 722 were affected (*11*). In 1979 and 1983, several small outbreaks of Getah virus infection occurred among unvaccinated horses at several facilities other than the Miho Training Center. Why an outbreak occurred again in 2014 remains unclear. We have no data on the vector mosquitoes or the climatic conditions in this region. Among the affected horses, eight 2-year-old horses had received the initial vaccination just 1–4 days before disease onset. Clearly, the vaccine against Getah virus could not provide some of the affected horses with sufficient protective immunity. The phylogenetic analyses showed that the currently circulating viruses differ genetically from the MI-110 vaccine strain isolated in 1978. From these results, we infer that this outbreak might be partly attributable to the antigenic differences between the vaccine strain and the currently circulating strain. Serologic studies of the current virus and the vaccine strain are in progress.

## References

[1] Griffin DE. Alphaviruses. In: Knipe DM, Howley PM, editors. Fields virology. Philadelphia: Lippincott Williams and Wilkins; 2013. p. 651–86.

[2] Fukunaga Y, Kumanomido T, Kamada M. Getah virus as an equine pathogen. Vet Clin North Am Equine Pract. 2000;16:605–17 . 11219353 10.1016/s0749-0739(17)30099-8

[3] Yago K, Hagiwara S, Kawamura H, Narita M. A fatal case in newborn piglets with Getah virus infection: isolation of the virus. Nippon Juigaku Zasshi. 1987;49:989–94. 10.1292/jvms1939.49.989 2828741

[4] Izumida A, Takuma H, Inagaki S, Kubota M, Hirahara T, Kodama K, Experimental infection of Getah virus in swine. Nippon Juigaku Zasshi. 1988;50:679–84. 10.1292/jvms1939.50.679 3210480

[5] Li XD, Qiu FX, Yang H, Rao YN, Calisher CH. Isolation of Getah virus from mosquitos collected on Hainan Island, China, and results of a serosurvey. Southeast Asian J Trop Med Public Health. 1992;23:730–4 . 1338481

[6] Brown CM, Timoney PJ. Getah virus infection of Indian horses. Trop Anim Health Prod. 1998;30:241–52. 10.1023/A:1005079229232 9760716

[7] Nemoto M, Yamanaka T, Bannai H, Tsujimura K, Kondo T, Matsumura T. Development and evaluation of a reverse transcription loop-mediated isothermal amplification assay for H3N8 equine influenza virus. J Virol Methods. 2011;178:239–42. 10.1016/j.jviromet.2011.07.015 21907240

[8] Wekesa SN, Inoshima Y, Murakami K, Sentsui H. Genomic analysis of some Japanese isolates of Getah virus. Vet Microbiol. 2001;83:137–46. 10.1016/S0378-1135(01)00417-5 11557154

[9] Lawrence GL, Gilkerson J, Love DN, Sabine M, Whalley JM. Rapid, single-step differentiation of equid herpesviruses 1 and 4 from clinical material using the polymerase chain reaction and virus-specific primers. J Virol Methods. 1994;47:59–72. 10.1016/0166-0934(94)90066-3 8051234

[10] Balasuriya UB, Leutenegger CM, Topol JB, McCollum WH, Timoney PJ, MacLachlan NJ. Detection of equine arteritis virus by real-time TaqMan reverse transcription-PCR assay. J Virol Methods. 2002;101:21–8. 10.1016/S0166-0934(01)00416-5 11849680

[11] Kamada M, Ando Y, Fukunaga Y, Kumanomido T, Imagawa H, Wada R, Equine Getah virus infection: isolation of the virus from racehorses during an enzootic in Japan. Am J Trop Med Hyg. 1980;29:984–8 . 6254385 10.4269/ajtmh.1980.29.984

[12] Imagawa H, Ando Y, Kamada M, Sugiura T, Kumanomido T, Fukunaga Y, Sero-epizootiological survey on Getah virus infection in light horses in Japan. Nippon Juigaku Zasshi. 1981;43:797–802. 10.1292/jvms1939.43.797 6283217

[13] Tamura K, Peterson D, Peterson N, Stecher G, Nei M, Kumar S. MEGA5: molecular evolutionary genetics analysis using maximum likelihood, evolutionary distance, and maximum parsimony methods. Mol Biol Evol. 2011;28:2731–9. 10.1093/molbev/msr121 21546353 PMC3203626

[14] Tajima S, Kotaki A, Yagasaki K, Taniwaki T, Moi ML, Nakayama E, Identification and amplification of Japanese encephalitis virus and Getah virus propagated from a single porcine serum sample: a case of coinfection. Arch Virol. 2014;159:2969–75. 10.1007/s00705-014-2152-x 24986716

